# Downregulation and Hypermethylation of Vitamin D Receptor in Lumbar Disc Degeneration

**DOI:** 10.3390/ijms26073226

**Published:** 2025-03-30

**Authors:** Ladawan Vajarintarangoon, Worawat Limthongkul, Weerasak Singhatanadgige, Vit Kotheeranurak, Wicharn Yingsakmongkol, Thananya Thongtan, Sinsuda Dechsupa, Sittisak Honsawek

**Affiliations:** 1 Center of Excellence in Osteoarthritis and Musculoskeleton, Department of Biochemistry, Faculty of Medicine, Chulalongkorn University, King Chulalongkorn Memorial Hospital, Thai Red Cross Society, Bangkok 10330, Thailand; ladawan.vaj@gmail.com (L.V.); thananyathongtan@gmail.com (T.T.); sinsuda.dech@gmail.com (S.D.); 2 Center of Excellence in Biomechanics and Innovative Spine Surgery, Department of Orthopaedics, Faculty of Medicine, Chulalongkorn University, King Chulalongkorn Memorial Hospital, Thai Red Cross Society, Bangkok 10330, Thailand; dr_worawat@hotmail.com (W.L.); dr.weerasaks@gmail.com (W.S.); vitinspine@gmail.com (V.K.); wicharn707@gmail.com (W.Y.)

**Keywords:** vitamin D receptor, expression, methylation, severity, lumbar disc degeneration

## Abstract

Lumbar disc degeneration (LDD) is a common musculoskeletal disorder that leads to chronic pain and functional impairment. Recent studies have suggested that the vitamin D receptor (VDR) plays a key part in regulating matrix metabolism, inflammation, and apoptosis in intervertebral discs (IVDs). The objective of this study was to examine cytokine expression and DNA methylation status of the VDR gene in blood leukocytes and lumbar disc tissues from patients with varying degrees of LDD severity. We aimed to explore correlations between VDR expression, methylation status, and clinical parameters such as pain intensity and functional disability. We conducted a prospective case-control study including 50 participants 35 LDD patients and 15 lumbar disc herniation (LDH) controls. Blood and lumbar disc tissue samples were collected for RNA and DNA extraction, followed by quantitative real-time PCR for gene expression and methylation-specific polymerase chain reaction for VDR promoter methylation analysis. Serum and nucleus pulposus (NP) VDR protein levels were measured using enzyme-linked immunosorbent assay. Clinical parameters, including pain intensity (NRS) and functional disability (ODI), were assessed. LDD patients exhibited significantly lower VDR mRNA expression in both blood leukocytes and NP tissue compared to controls (*p* < 0.05). LDD patients had significantly greater serum TNF-α levels than controls (*p* < 0.001); however, serum IL-1β levels were not different between two groups. Serum VDR protein levels were elevated in LDD patients (*p* = 0.016), whereas NP VDR protein was significantly reduced in the LDD group (*p* = 0.013). VDR promoter methylation was significantly higher in both the blood and NP tissue of LDD patients compared to controls (*p* < 0.001). Additionally, higher VDR promoter methylation in blood was correlated with advanced disc degeneration (*p* < 0.05), while NP methylation was associated with all grades of degeneration (*p* < 0.001). Serum VDR protein levels were inversely correlated with pain intensity (*r* = −0.39, *p* = 0.02), while NP VDR levels positively correlated with NRS scores (*r* = 0.43, *p* = 0.01). Aberrant VDR expression and increased promoter methylation are associated with LDD severity. Dysregulated VDR signaling, potentially mediated by DNA methylation, may play a critical role in the pathophysiology of LDD. These findings suggest that VDR could be a novel biomarker reflecting disease severity and a potential therapeutic target for managing LDD.

## 1. Introduction

Lumbar disc degeneration (LDD) is a pervasive musculoskeletal disorder characterized by the progressive deterioration of intervertebral discs (IVDs) in the lumbar spine, often resulting in debilitating back pain and functional impairment [1]. A number of etiological factors act as primary initiators of the abnormal production of catabolic molecules by IVD cells [2]. These factors include aging, genetic susceptibility, smoking, illness, aberrant biomechanical loading, and inadequate nutrient supply [2,3]. Inflammatory cytokines, particularly tumor necrosis factor-α (TNF-α) and interleukin-1β (IL-1β), are fundamental contributors to the pathophysiology of lumbar disc degeneration [4]. Despite its prevalence and clinical significance, the precise molecular mechanisms underlying LDD pathogenesis have yet to be fully elucidated.

Among the factors implicated in the pathogenesis of LDD, the role of the vitamin D receptor (VDR) has garnered increasing attention in recent years. The VDR, a nuclear hormone receptor that mediates the biological effects of vitamin D, has emerged as a potential player in LDD pathogenesis [5]. Vitamin D and its receptor have been implicated in various cellular processes relevant to disc homeostasis, including matrix metabolism, inflammation, and apoptosis, all of which are disrupted in LDD [5,6,7]. Several lines of evidence suggest that the dysregulation of VDR signaling exacerbates these disruptions, contributing to extracellular matrix degradation and heightened inflammatory responses. Additionally, VDR regulates oxidative stress and autophagy, which are essential for maintaining cellular function in the intervertebral disc under both normal and pathological conditions [6,8]. Moreover, vitamin D deficiency has been associated with various musculoskeletal disorders, raising questions regarding its potential involvement in LDD.

In recent years, there has been growing interest in the role of epigenetic modifications, particularly DNA methylation, in the development and progression of LDD [9]. DNA methylation, an epigenetic process involving the addition of methyl groups to cytosine residues within CpG dinucleotides, plays a crucial role in regulating gene expression and chromatin structure. Vitamin D and aberrant DNA methylation patterns have been implicated in various pathological conditions, including cancer, aging, and musculoskeletal disorders [10,11]. Despite the individual roles of DNA methylation and VDR in LDD pathogenesis, the interplay between DNA methylation and VDR expression in disease pathophysiology remains poorly characterized.

The purpose of this study was to investigate the cytokine expression and DNA methylation status of the VDR gene in blood leukocytes and lumbar disc tissues obtained from patients with varying degrees of LDD severity. We hypothesized that aberrant DNA methylation in the regulatory regions of VDR contributes to dysregulated VDR expression, which may exacerbate LDD pathophysiology. Furthermore, we aimed to explore potential associations between VDR expression and methylation status and clinical parameters such as pain intensity, functional disability, and radiographic features of LDD. By elucidating the molecular underpinnings of VDR dysregulation in LDD, this study holds promise for identifying novel biomarkers and therapeutic targets for the early diagnosis and management of this debilitating condition.

## 2. Results

### 2.1. Baseline Characteristics

The current study recruited a total of 50 subjects: 35 LDD patients and 15 lumbar disc herniation (LDH) controls, with a mean age of 66.43 ± 1.30 vs. 38.13 ± 3.04 years, respectively (*p* < 0.001). There were no significant differences in sex ratio, body mass index (BMI), and numerical rating scale (NRS) pain scores (Table 1). LDD patients had significantly greater serum TNF-α levels than controls (*p* < 0.001); however, serum IL-1β levels were not significantly different between LDD patients and controls (Table 1). The LDD subjects demonstrated a considerably higher median Oswestry Disability Index (ODI) score (42.22%, IQR: 35.56–54.44%) compared to the controls (13.33%, IQR: 8.89–22.22%) (*p* < 0.001). Nearly half (48.6%) of the LDD participants experienced mild disability (D1), while 34.30% reported moderate disability (D2), and 17.10% suffered from severe disability (D3). In contrast, all participants in the control group were categorized under the mild disability category, according to the ODI.

Interestingly, despite similar pain levels, the LDD group experienced a significantly greater functional burden due to their disability. Analysis of the LDD cohort using the Pfirrmann grading system for disc degeneration revealed a predominance of advanced stages: 25.70% (*n* = 9) with grade 3, 34.30% (*n* = 12) with grade 4, and 40.00% (*n* = 14) with grade 5 degeneration (Table 2). This finding suggests a potential association between the severity of disc degeneration and functional limitations in the LDD group.

### 2.2. Blood VDR mRNA Downregulation in LDD Patients

**Figure 1:**
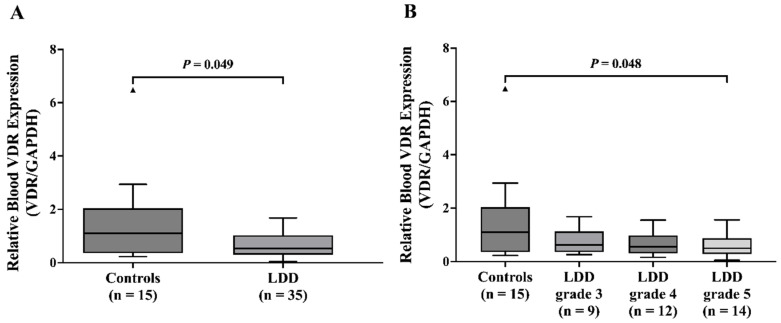
Relative blood VDR mRNA expression in (**A**) controls and LDD patients and (**B**) controls and LDD patients with various Pfirrmann grades. The dark triangles indicate outliers.

Analysis of VDR mRNA expression in blood leukocytes revealed a trend toward decreased expression in LDD patients compared to controls (*p* = 0.049). The median VDR/GAPDH ratio was significantly lower in the LDD group (0.53, IQR: 0.31–0.95) than in the control group (1.10, IQR: 0.38–1.84) (Figure 1A). This finding suggests a potential downregulation of VDR in the peripheral blood cells of LDD patients. The control group exhibited significantly higher VDR expression compared to LDD patients with Pfirrmann grade 5 (*p* = 0.048) (Figure 1B), However, no significant association in VDR expression was observed among LDD patients with various Pfirrmann grades (*p* = 0.201).

### 2.3. NP VDR mRNA Downregulation in LDD Patients

**Figure 2:**
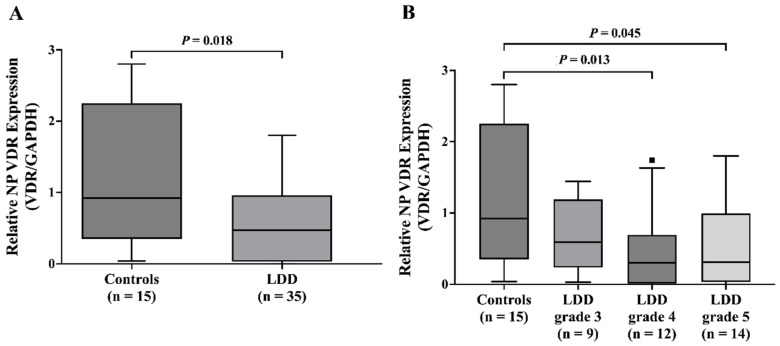
Relative NP VDR mRNA expression in (**A**) controls and LDD patients and (**B**) controls and LDD patients with grade 3, 4, and 5. The dark square indicates outlier.

Similar to blood VDR, the analysis of NP VDR expression also showed a trend toward lower levels in the LDD group compared to controls (*p* = 0.018). The median VDR/GAPDH ratio was 0.47 (IQR: 0.04–0.96) in the LDD group and 0.92 (IQR: 0.54–2.24) in the control group (Figure 2A). The control group exhibited significantly greater NP VDR expression compared to LDD patients with advanced disc degeneration (LDD grades 4 and 5; *p* = 0.013 and *p* = 0.045, respectively) (Figure 2B).

### 2.4. Elevated Serum VDR Protein Levels in LDD Patients

**Figure 3:**
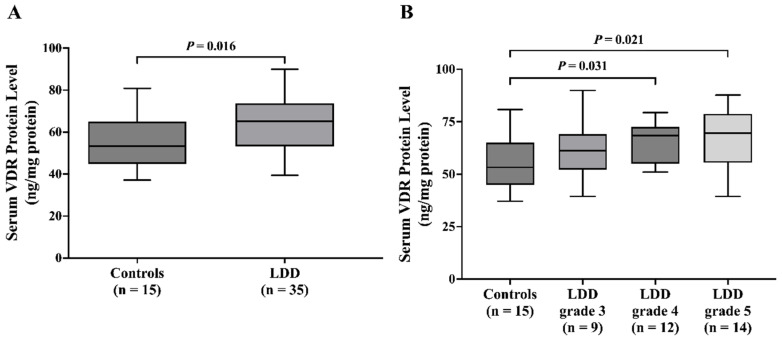
Serum VDR protein level in (**A**) controls and LDD patients and (**B**) controls and LDD patients with grade 3, 4, and 5.

Analysis of serum VDR protein levels revealed a significant increase in the LDD group compared to controls (*p* = 0.016). The median serum VDR protein level in the LDD group was 65.20 ng/mg (IQR: 53.47–73.15), while the control group had a median of 53.28 ng/mg (IQR: 46.24–62.86) (Figure 3A). Consistent with VDR mRNA expression in blood leukocytes, serum VDR protein levels did not show a significant association with the severity of LDD Pfirrmann grading (*p* = 0.076). Additionally, the control group displayed significantly lower serum VDR protein levels than the LDD patients with advanced disc degeneration stages (LDD grades 4 and 5; *p* = 0.031 and 0.021, respectively) (Figure 3B).

### 2.5. Decreased NP VDR Protein Levels in LDD Patients

**Figure 4:**
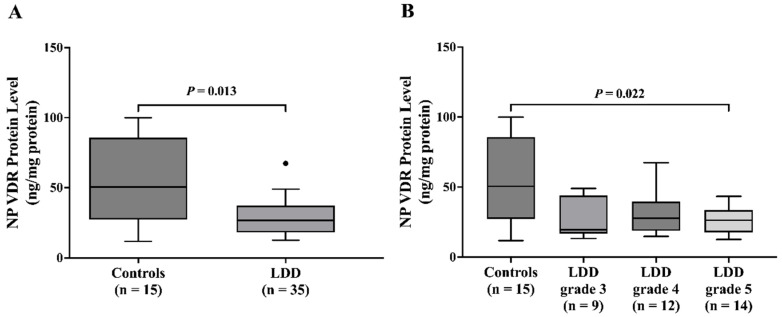
NP VDR protein level in (**A**) controls and LDD patients and (**B**) controls and LDD patients with grade 3, 4, and 5. The dark dot indicates outlier.

Analysis of NP VDR protein levels also showed a significant decrease in the LDD group compared to controls (*p* = 0.013). The median NP VDR protein level in the LDD group was 26.68 ng/mg (IQR: 18.34–36.70), whereas the control group had a median level of 50.51 ng/mg (IQR: 29.56–85.24) (Figure 4A). In addition, the control group exhibited significantly higher NP VDR protein levels than the LDD patients with advanced disc degeneration stages (LDD grade 5; *p* = 0.022, respectively) (Figure 4B).

### 2.6. Increased VDR Promoter Methylation in LDD Patients

**Figure 5:**
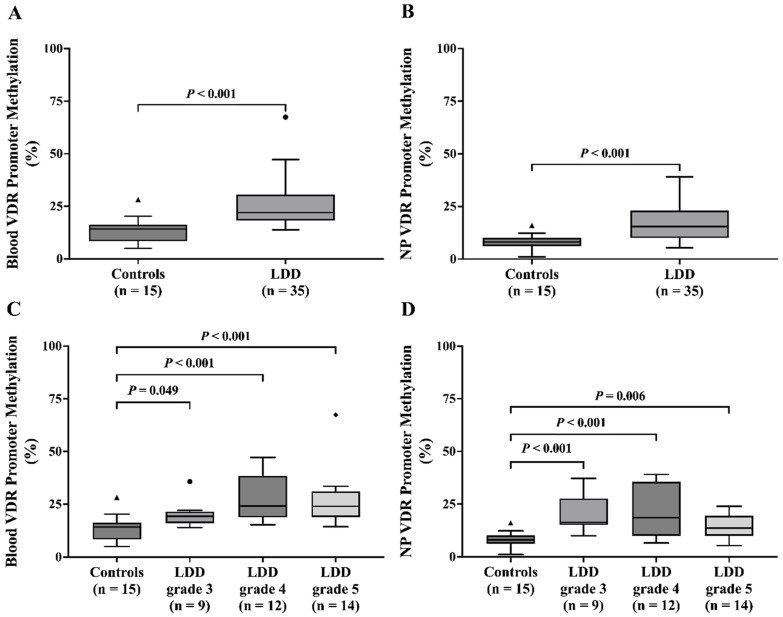
Blood VDR promoter methylation in (**A**) blood leukocytes and (**B**) NP of controls and LDD patients, blood VDR promoter methylation in (**C**) blood leukocytes and (**D**) NP of controls and LDD patients with grade 3, 4, and 5. The dark different shapes indicate outliers.

As shown in Figure 5A,B, VDR promoter methylation was significantly higher in LDD patients than in the controls (blood: 22.02% vs. 14.18%, *p* < 0.001; NP: 15.52% vs. 8.16%, *p* < 0.001). Analysis based on Pfirrmann grade indicated a possible association between higher blood VDR methylation and advanced degeneration stages (Figure 5C). However, NP tissue exhibited a direct relationship, with significant differences observed between controls and all Pfirrmann grades (*p* < 0.001) (Figure 5D).

### 2.7. Correlation Between VDR Protein Expression NRS Scores

Spearman’s rank correlation analysis revealed statistically significant associations between VDR protein expression and NRS scores. Serum VDR levels demonstrated a negative correlation with NRS (*r* = −0.39, *p* = 0.02), suggesting a potential inverse relationship between higher serum VDR levels and lower NRS scores. Conversely, NP VDR levels exhibited a positive correlation with NRS (*r* = 0.43, *p* = 0.01), indicating a possible association between elevated NP VDR and increased NRS scores.

## 3. Discussion

The analysis of demographic data revealed a significant age difference between the LDD and control groups, with the LDD group having a higher mean age. This finding aligns with the established knowledge that the prevalence of disc degeneration increases with age due to the gradual breakdown of intervertebral disc (IVD) components [12]. The observed age difference between the LDD and control groups highlights the critical role of age in LDD development. As individuals age, the IVDs undergo degeneration characterized by a loss of proteoglycans, reduced water content, and weakened structural integrity [13]. These age-related changes make the IVDs more vulnerable to mechanical stress and injury, ultimately contributing to LDD development. While gender and overall BMI did not differ significantly between the groups, weight may still play a role. The LDD group had a higher average BMI, classifying them within the obese class I category. This observation is consistent with the previous research by Liuke M et al. [14], which suggests a potential link between elevated BMI and an increased risk of LDD.

In this study, LDD patients and controls reported moderate to severe pain (NRS scores). However, LDD patients displayed greater limitations in daily activities (ODI scores). In line with the findings of Middendorp et al. [15], who assessed ODI scores in LDD patients at L4/L5 and L5/S1, our study demonstrates a positive association between the severity of disc degeneration and ODI scores. We also observed a significant decrease in VDR mRNA levels within both blood leukocytes and NP tissues of the LDD group. These findings align with prior observations by Taha et al. [16] who reported similar reductions in blood VDR levels in LDD patients compared to healthy controls. Furthermore, a recent study showed low VDR expression in peripheral blood mononuclear cells in osteoporosis patients [17].

Intriguingly, serum VDR levels were significantly higher in the LDD group than in the control group. Subgroup analysis by Pfirrmann grade showed an association between higher circulating VDR and advanced disc degeneration (Pfirrmann grades 4 and 5). This finding aligns with the potential for higher VDR to exert a protective effect, possibly through modulation of pain-sensing and processing or inflammatory pathways [18]. In contrast, NP VDR protein levels were significantly lower in LDD patients compared to controls. This finding aligns with the prior research by Yang Q et al. [19] who demonstrated a substantial reduction in VDR expression within the LDD group compared to controls. The explanation for conflicting results could be that elevated serum VDR might reflect compensatory mechanisms, possibly indicating an adaptive response to the dysregulation of VDR signaling at the tissue level. However, this increase did not correlate significantly with the severity of LDD, suggesting that serum VDR levels might not be directly reflective of the local disc pathology but could instead be influenced by other systemic factors related to the disease. Further studies are needed to elucidate the mechanisms underlying this elevation and its potential implications for disease progression and treatment.

This study explored the potential role of VDR promoter methylation, a marker of gene silencing, in LDD. The analysis revealed significantly increased VDR promoter methylation levels in both blood and NP tissue of LDD patients compared to controls. Our findings regarding VDR promoter methylation in LDD align with a recent study [20], particularly those affecting the VDR methylation. VDR plays a crucial role in osteogenic differentiation, osteogenesis, and bone remodeling. This observed methylation pattern suggests a potential mechanism contributing to the downregulation of VDR expression in LDD. Moreover, age-associated hypermethylation of CpG sites provides additional evidence that DNA methylation increases with aging, potentially impairing the expression of genes like VDR that are critical for cellular homeostasis and tissue function [21]. In addition, blood VDR promoter methylation was significantly associated with higher Pfirrmann grades, suggesting a potential link between VDR promoter methylation and the severity of disc degeneration. This is consistent with the findings of Maruthai K et al., who observed elevated VDR promoter methylation in tuberculosis patients compared to healthy controls [22].

Inflammatory cytokines, especially TNF-α and IL-1β, are known as key mediators in the progression of disc degeneration [4,23]. TNF-α and IL-1β contribute to disc cell aging, extracellular matrix degradation, and disc degeneration [23]. Previous studies showed that TNF-α and IL-1β are found to be highly expressed in degenerative discs [23,24]. TNF-α and IL-1β are involved in initiation and progression of disc degeneration by regulating the inflammatory response, senescence, apoptosis, and matrix destruction [23,25]. The current study also demonstrated that serum TNF-α was significantly higher in LDD patients than in LDH controls. However, serum IL-1β levels did not significantly differ between these two groups. Several potential explanations for this observation warrant discussion. First, IL-1β is primarily produced and acts locally within the disc and surrounding tissues. Both LDD and LDH involve localized inflammatory processes, where IL-1β contributes to matrix degradation, disc cell apoptosis, and nociceptive signaling. However, systemic circulation may not accurately reflect these local inflammatory changes, leading to undetectable differences in serum IL-1β levels. Second, given that IL-1β plays a central role in both diseases, it is possible that its systemic levels may be comparable. Moreover, the different stages of disease progression among patients may influence serum IL-1β levels. For instance, LDH may represent an acute or subacute phase of disc pathology, whereas LDD is often a chronic, progressive condition. Variability in disease duration and severity may result in fluctuating IL-1β levels, ultimately leading to no significant differences in cross-sectional analyses. Lastly, the sensitivity of detection immunoassays also merits consideration. More sensitive methods, such as multiplex cytokine assays or mass spectrometry, may be required to detect the subtle differences between patient groups.

The observed increase in serum VDR levels, alongside decreased VDR expression in NP tissue, highlights a complex interplay between systemic and local regulation of VDR. Elevated serum VDR levels may reflect a compensatory response to local dysregulation, as decreased VDR expression in NP tissue could trigger systemic adjustments aimed at mitigating its functional loss. Additionally, VDR promoter hypermethylation observed in NP tissue likely contributes to the localized downregulation of VDR, whereas systemic factors, such as inflammation or vitamin D metabolism, may drive increased serum VDR levels. This duality underscores the multifaceted role of VDR in LDD pathophysiology.

One of the major strengths of this study is its comprehensive approach to exploring the relationship between VDR expression and LDD. The inclusion of multiple sample types—blood leukocytes, NP tissue, and serum—provides a holistic view of VDR activity in LDD patients, allowing for a thorough analysis of both gene expression and protein levels. Moreover, the use of the Pfirrmann grading system to categorize disc degeneration stages adds a robust, clinically relevant framework for assessing the severity of degeneration and its potential association with VDR expression. Finally, the results reveal novel findings such as increased serum VDR protein levels in LDD patients and a significant increase in VDR promoter methylation, which contributes to advancing our understanding of molecular mechanisms underlying disc degeneration.

There are several limitations to consider in this study. First, the sample size was relatively small, with only 50 participants, which may limit the generalizability of the results. The unequal distribution of LDD patients and controls further compounds this limitation, potentially introducing bias. Second, this study’s cross-sectional design does not allow for the establishment of causal relationships between VDR expression and LDD severity. Third, this study also only focused on the blood and NP tissue samples, which may not fully capture the systemic effects of VDR downregulation, or the impact of other tissues involved in disc degeneration. Furthermore, while this study measured VDR expression and promoter methylation, it did not determine other potential confounding factors such as genetic variability, diet, or environmental influences that could contribute to LDD progression. Finally, this study did not explore potential variations in VDR expression across different ethnic groups or the role of other related vitamin D pathways, which may provide additional insights into the pathophysiology of LDD.

In conclusion, this study provides novel insights into the role of VDR expression in LDD, highlighting significant alterations in VDR mRNA and protein levels in both peripheral blood and NP tissues. The downregulation of VDR in the NP and blood leukocytes, along with increased promoter methylation, suggests a potential epigenetic mechanism contributing to the pathophysiology of LDD. Furthermore, the complex relationship between VDR expression and pain scores offers a compelling avenue for further research into VDR as a therapeutic target. These findings underscore the need for continued exploration of VDR’s role in musculoskeletal diseases, which may lead to new diagnostic and treatment strategies for LDD patients.

## 4. Materials and Methods

### 4.1. Study Participants

The research protocol was approved by the Institutional Review Board (IRB) of the Faculty of Medicine, Chulalongkorn University (IRB No. 0219/66). Written informed consent was obtained from all participants prior to their enrollment in this study.

The prospective case–control study was conducted between May 2023 and December 2023. A total of 50 participants were included: 35 patients with LDD and 15 patients with LDH as the control group. All participants were recruited from the Department of Orthopaedics at King Chulalongkorn Memorial Hospital. The inclusion criteria were low back pain and radiculopathy related to either LDD or LDH, diagnosed using lumbar magnetic resonance imaging (MRI). Participants were excluded if they had a history of lumbar surgery, epidural steroid injections within the last 6 months, trauma, recent infections, congenital anomalies, osteoporosis, cancer, vertebral fractures, spinal deformities, or were pregnant or breastfeeding at the time of recruitment.

### 4.2. Radiographic Severity Assessment

MRI scans of T2-weighted spin-echo images of the lumbosacral spine were examined to assess the severity of degeneration. The Pfirrmann grading system [26], which ranges from grades 1 to 5, was utilized for classification. Grade 1 denotes a healthy disc characterized by a bright, hyperintense white signal intensity, clear differentiation between the nucleus pulposus (NP) and annulus fibrosus (AF), and normal disc height. Conversely, grade 5 indicates a hypointense black signal intensity with no distinct boundary between the NP and AF, accompanied by disc space collapse. Grades 2 and 3 represent mild and moderate changes, respectively, while grades 4 and 5 indicate severe changes in disc morphology. In this study, LDD patients were included if they had a Pfirrmann grade of ≥3.

### 4.3. Self-Report Assessment

This study employed a self-report assessment methodology, utilizing questionnaires to evaluate various aspects of patients’ conditions. Specifically, pain levels were quantified using numerical rating scales (NRSs) upon admission, prior to surgical procedures. Patients were instructed to indicate their current pain intensity on a numerical scale ranging from 0 (no pain) to 10 (severe pain), with specific guidance provided on the interpretation of each numerical value. Additionally, activity limitation was assessed through the modified Oswestry Disability Index (ODI) [27] questionnaire, which evaluated pain severity, self-care abilities, mobility, and social participation. The ODI scores were then converted into percentages to facilitate the interpretation of disability levels, which were categorized into five distinct ranges based on the degree of activity limitation. In this study, the ODI was categorized into three groups based on severity, with higher scores indicating greater disability: mild disability (0–40%), moderate disability (41–60%), and severe disability (61–100%).

### 4.4. Blood and Tissue Collection

Blood samples were obtained through venipuncture using ethylenediamine tetra-acetic acid (EDTA) and clotted blood collection tubes. After centrifugation at 4000 rpm for 10 min at 25 °C, plasma and the buffy coat were separated from the EDTA tubes, while serum samples were acquired from the clotted blood tubes. NP tissue samples were procured from patients who underwent surgery using different surgical procedures based on the underlying pathology. To ensure accurate NP sampling, tissue was carefully identified during surgery by the surgeon based on its anatomical characteristics, distinct gelatinous texture, and surgical landmarks. Any fibrotic or annular components were minimized to reduce contamination with non-NP tissue. Meticulous care was taken during both the collection and preservation of samples in microcentrifuge tubes. RNAprotect Tissue Reagent (QIAGEN, Hilden, Germany) was then added to each tube post-collection to preserve RNA integrity within the tissue samples.

### 4.5. RNA Isolation and cDNA Synthesis

Total RNA was extracted from whole blood and NP samples using the GF-1 Total RNA Extraction Kit (Vivantis, Subang Jaya, Selangor, Malaysia). The purity and concentration of RNA were measured using a Nanodrop 2000 spectrophotometer (Thermo Fisher Scientific, Waltham, MA, USA). Complementary DNA (cDNA) was synthesized from 250 ng of total RNA according to the manufacturer’s instructions using the cDNA Synthesis Kit (Biotechrabbit, Berlin, Germany), and subsequently analyzed using quantitative real-time polymerase chain reaction (qPCR).

### 4.6. DNA Isolation

Genomic DNA was extracted from blood samples collected in EDTA-containing tubes and from NP samples obtained from participants using the GF-1 Blood DNA Extraction Kit (Vivantis, Subang Jaya, Selangor, Malaysia) and the GF-1 Tissue DNA Extraction Kit (Vivantis, Subang Jaya, Selangor, Malaysia), respectively. The purity and concentration of DNA were assessed using a Nanodrop 2000 spectrophotometer (Thermo Fisher Scientific, Waltham, MA, USA). Subsequently, the extracted DNA was stored at −80 °C for further analysis.

### 4.7. Bisulfite Conversion

For bisulfite conversion, genomic DNA was subjected to the EZ DNA Methylation-Lightning Kit (ZymoResearch Inc., Irvine, CA, USA), following the manufacturer’s guidelines. Specifically, 100 ng of genomic DNA from both blood and NP underwent bisulfite conversion to discriminate between methylated and unmethylated cytosine (C). After conversion, the bisulfite-treated DNA was eluted with 10 μL of elution buffer and stored at −20 °C until required for subsequent analysis. Commercially available fully methylated (M) and fully unmethylated (U) bisulfite-converted control DNAs were obtained from EpiTect PCR Control DNA Set (QIAGEN, Hilden, Germany). These M and U control DNAs were combined to produce a panel of DNA standards with varying methylation levels (100%, 75%, 50%, 25%, 10%, 5%, 0%). This panel of standards was then utilized in PCR amplification protocols.

### 4.8. Primer Design

Genomic sequence of the VDR gene was acquired from the National Center for Biotechnology Information (NCBI) and Ensembl databases. Primer design for VDR amplification was facilitated using the Primer-BLAST tool, with glyceraldehyde-3-phosphate dehydrogenase (GAPDH) serving as the internal control [28]. Additionally, the consensus DNA sequence of the promoter was retrieved from the Eukaryotic Promoter Database (EPD). Subsequently, the CpG island Finder tool was used to precisely identify the locations of CpG sites and CpG Islands within the promoter sequence. Methylation specific-PCR (MS-PCR) primers were then designed using the MethPrimer tool [29] (Table 2). Two separate PCR reactions were performed, each aimed at amplifying either methylated (M) or unmethylated (U) specific regions.

### 4.9. Quantification of VDR Gene Expression

Quantification of VDR gene expression via qPCR followed a meticulous procedure. The primer sets employed in this study are listed in Table 1. Amplification of both housekeeping and target genes from cDNA was conducted in a total reaction volume of 20 μL. This reaction mixture consisted of 2 μL of cDNA, 10 μL of SYBR green master mix (Biotechrabbit, Berlin, Germany), 0.4 μL of each primer (200 nM), and 7.2 μL of RNase-free water. The amplification was carried out using the Applied Biosystem 7500 Fast Real-Time PCR System (Thermo Fisher Scientific, Foster City, CA, USA). PCR cycling conditions included an initial denaturation at 95 °C for 2 min, followed by 40 cycles at 95 °C for 30 s, 58 °C for 30 s, and a final extension at 72 °C for 30 s. Relative VDR gene expression levels were calculated using the equation 2^(−∆∆Ct)^, where ∆Ct represents the difference in cycle threshold (Ct) values between the target and reference genes. The ∆∆Ct parameter quantified the variation between the ∆Ct of the target sample and the mean ∆Ct of the control samples.

### 4.10. Methylation-Specific PCR (MS-PCR)

Two distinct PCR reactions were performed, each specifically targeting either M or U for PCR amplification. The MS-PCR primers utilized in this study are meticulously detailed in Table 2. A 20 μL PCR reaction mixture was prepared, comprising 2 μL of bisulfite-converted DNA, 1 μL of each forward and reverse primer (10 μM) for M or U-specific primers (in separate reactions), 8 μL of ZymoTaq PreMix (Zymo Research, Irvine, CA, USA), and 8 μL of nuclease-free water. The PCR cycling protocol started with an initial denaturation at 95°C for 10 min, followed by 35 cycles of denaturation at 95 °C for 30 s, annealing at 56 °C (M) or 56.5 °C (U) for 35 s, and extension at 72 °C for 30 s, with a final extension at 72 °C for 7 min. The resulting products were then stored at −20 °C until further utilization.

### 4.11. Assessment of VDR Promoter Methylation

The PCR-amplified products were separated by 2% agarose gel electrophoresis. The presence or absence of both M- and U-specific MS-PCR products, alongside band intensity, was documented. Band density was subsequently analyzed using the ImageJ Software version 1.54 [30] and compared to methylation control standards. Methylation scoring analysis was then conducted to categorize the distribution of methylation levels as a percentage (%) based on the intensity of the PCR bands.

The MS-PCR products were classified into four groups: unmethylated (0–10% band intensity), weakly methylated (10–40% band intensity), moderately methylated (40–70% band intensity), and strongly methylated (70–100% band intensity).

The same methylation scoring method was applied to control DNA sets, and samples were categorized based on band intensity. To minimize potential bias, strict PCR conditions were maintained, and results were interpreted accordingly. The MS-PCR experiment was repeated to ensure consistent band intensity, and the percentage methylation level distribution of the VDR gene in each sample was subsequently calculated.

### 4.12. Total Protein Extraction

NP tissue protein extraction began by promptly placing samples in pre-chilled tubes and homogenizing them in ice-cold RIPA buffer (Abcam, Cambridge, UK) containing protease inhibitors. After centrifugation at low speed and 4 °C, a protein-enriched supernatant was obtained. Total protein concentration was determined using the bicinchoninic acid (BCA) method, and aliquots were stored at −80 °C.

### 4.13. Enzyme-Linked Immunosorbent Assay

Serum LDD and controls samples were analyzed to determine the concentrations of TNF-α (DY210-05) and IL-1β (DY201-05) using enzyme-linked immunosorbent assay (ELISA) kit (R&D Systems, Inc., Minneapolis, MN, USA). According to the manufacturer’s protocol, the capture antibodies were diluted to the working concentration and immediately used to coat a 96-well microplate. The plate was sealed and incubated overnight at room temperature. Following incubation, the wells were aspirated, washed three times, then blocked by reagent diluent to each well, and incubated at room temperature for at least one hour. Recombinant human TNF-α and IL-1β standards, and serum samples were diluted and dispensed into microplates with specific antibodies. The plates were incubated at room temperature for 2 h, after which the wells were washed four times with a wash buffer to remove unbound components. Subsequently, TNF-α and IL-1β conjugates were added to each well, and the plates were incubated for 2 h at room temperature. After another series of four washes, a substrate solution was introduced to each well, followed by a 20 min incubation at room temperature in the absence of light. The reaction was then halted using a stop solution, and absorbance was measured at 450 nm with optical correction performed at 540 nm using a spectrophotometer. The serum concentrations of TNF-α and IL-1β were quantified using a standard optical density concentration curve.

Serum VDR and NP VDR levels were assessed using a commercially available human VDR ELISA kit (ELK2396, ELK Biotechnology, Denver, CO, USA). Following the manufacturer’s protocol, both standards and samples were added to microplate wells, then incubated at 37 °C for antibody binding. After washing, biotinylated antibody and streptavidin-horseradish peroxidase solutions were added to the wells. The 3, 3′, 5, 5′-tetramethylbenzidine (TMB) substrate induced color change, and absorbance readings were taken at 450 nm. Standard curves were constructed to calculate VDR protein levels from sample absorbance values, providing insights into protein expression levels. The detection range was from 0.63 ng/mL to 40 ng/mL.

### 4.14. Statistical Analysis

The statistical analysis was accomplished using SPSS Statistics version 22.0 (SPSS, Chicago, IL, USA) and GraphPad Prism version 9.0 (GraphPad Software, San Diego, CA, USA). The normality of the data distributions was assessed using the Shapiro–Wilk test with visual inspection of histograms and Q-Q plots. Quantitative data with a normal distribution were presented as mean ± standard error of the mean (SEM), while quantitative data with a non-normal distribution were presented as median (the first quartile; Q1–the third quartile; Q3). Differences in age, BMI, NRS, ODI, mRNA expression levels, and methylation status of VDR between the LDD and control groups were evaluated using unpaired Student’s *t*-test and Mann–Whitney *U* test, as appropriate. Gender differences were analyzed with the Chi-square test. Analysis of variance (ANOVA) or the Kruskal–Wallis test was employed for analyses involving more than two groups, contingent on data distribution. Correlations among variables were examined using Pearson’s correlation for normally distributed data and Spearman’s correlation for non-normally distributed data. A *p* < 0.05 was considered statistically significant.

## Figures and Tables

**Table 1:** Baseline characteristics of participants.

Characteristics	LDD	Controls	*p*-Value
*n*	35	15	
Age (years), mean ± SEM	66.43 ± 1.30	38.13 ± 3.04	**<0.001 ^a^**
Gender			0.558 ^b^
Female, *n*, %	24 (68.60%)	9 (60.00%)	
Male, *n*, %	11 (31.40%)	6 (40.00%)	
BMI (kg/m^2^), median (Q1–Q3)	25.51 (20.69–28.23)	23.66 (22.32–26.21)	0.958 ^c^
NRS (point), median (Q1–Q3)	7.00 (5.50–8.00)	6.00 (5.00–7.00)	0.288 ^c^
TNF-α (Q1–Q3)	34.33 (31.67–37.67)	14.67 (11.67–19.33)	**<0.001 ^c^**
IL-1β (Q1–Q3)	2.06 (1.71–2.92)	2.77 (2.62–3.34)	0.49 **^c^**
ODI (%), median (Q1–Q3)	42.22 (35.56–54.44)	13.33 (8.89–22.22)	**<0.001 ^c^**
D1, *n* (%)	17 (48.60%)	15 (100.00%)	—
D2, *n* (%)	12 (34.30%)	0 (0.00%)	—
D3, *n* (%)	6 (17.10%)	0 (0.00%)	—
Pfirrmann grade, *n*, %		—	—
grade 3	9 (25.70%)		
grade 4	12 (34.30%)		
grade 5	14 (40.00%)		

Significant results are shown in bold. ^a^ unpaired Student’s *t*-test; ^b^ Chi-square test; ^c^ Mann–Whitney *U* test. BMI: body mass index; NRS: numerical rating scale; ODI: Oswestry Disability Index; D1: mild disability; D2: moderate disability; D3: severe disability; SEM: standard error of the mean.

**Table 2:** Primer design.

			Sequence of Primer 5′-3′	Length	Tm (°C)
Primers for qPCR					
VDR		FW	ACCAGGATTCAGAGACCTCAC	21	62.0
		RW	AGTCTTGGTTGCCACAGGTC	20	62.8
GAPDH		FW	GTCTCCTCTGACTTCAACAGCG	22	58.9
		RW	ACCACCCTGTTGCTGTAGCCAA	22	62.7
Primers for MS-PCR					
VDR	M	FW	GATTTAGTTTTTTTGGGTGAGATTC	25	50.5
		RW	CCAACCTATTACTACTACAAAACGC	25	53.9
	U	FW	GATTTAGTTTTTTTGGGTGAGATTT	25	49.7
		RW	CAACCTATTACTACTACAAAACACC	25	51.3

qPCR, quantitative real-time polymerase chain reaction; MS-PCR, methylation-specific polymerase chain reaction; VDR, vitamin D receptor; GAPDH, glyceraldehyde-3-phosphate dehydrogenase; M, methylation; U, unmethylation; FW, forward; RW, reverse; Tm, melting temperature.

## Data Availability

All data are contained within the article.

## Abbreviations

AF	Annulus fibrosus
BMI	Body mass index
EDTA	Ethylenediamine tetra-acetic acid
GAPDH	Glyceraldehyde 3-phosphate dehydrogenase
IQR	Interquartile range
IVD	Intervertebral disc
LDD	Lumbar disc degeneration
LDH	Lumbar disc herniation
M	Methylation
MRI	Magnetic resonance imaging
MS-PCR	Methylation specific-polymerase chain reaction
NP	Nucleus pulposus
NRS	Numerical rating scale
ODI	Oswestry Disability Index
PCR	Polymerase chain reaction
qPCR	Quantitative real-time polymerase chain reaction
SEM	Standard error of the mean
Tm	Melting temperature
U	Unmethylation
VDR	Vitamin D receptor
